# Acute effects of proprioceptive neuromuscular facilitation stretching on rectus femoris muscle stiffness: a dose-response shear-wave elastography study

**DOI:** 10.3389/fphys.2024.1496825

**Published:** 2025-01-09

**Authors:** Sara Kranjc, Mojca Fink, Masatoshi Nakamura, Žiga Kozinc

**Affiliations:** ^1^ Faculty of Health Sciences, University of Primorska, Koper, Slovenia; ^2^ Faculty of Rehabilitation Sciences, Nishi Kyushu University, Kanzaki, Saga, Japan

**Keywords:** muscle elasticity, acute effects, dose-response, ultrasound elastography, stretching intervention

## Abstract

**Introduction:**

Proprioceptive neuromuscular facilitation (PNF) stretching is widely used to increase range of motion, but its underlying mechanisms are not well understood. This experimental, parallel group design study investigated the acute effects of PNF stretching on rectus femoris muscle stiffness and explored a potential dose-response relationship.

**Methods:**

Thirty healthy young adults (23 females, 7 males) were randomly assigned to either a PNF stretching group (n = 15; 22.96 ± 2.2 years) or a control group (n = 15; 23.3 ± 2.1 years). Rectus femoris stiffness was measured using shear-wave elastography (Resona 7, Mindray, China) at two locations (distal and proximal) before and after the second, fourth, and sixth sets of PNF stretching. The protocol involved six sets, each with three 10-s stretches and 5-s maximal contractions.

**Results:**

The results indicate that PNF stretching had no statistically significant effect on muscle stiffness, with no main effects of group (F = 0.05; *p* = 0.830) or time (F = 0.545; *p* = 0.653), and no significant interactions. However, the proximal location showed a substantially higher shear modulus compared to the distal location (F = 63.6; *p* < 0.001; η^2^ = 0.69), independent of group or time.

**Discussion:**

These findings highlight a location-specific difference in muscle stiffness that was unaffected by the intervention. In conclusion, PNF stretching did not acutely reduce rectus femoris stiffness compared to passive rest, regardless of the number of stretching sets performed. Further research is needed to understand the muscle-specific effects of PNF stretching.

## 1 Introduction

Stretching is often used in sports as part of the warm-up, with the goal of increasing joint range of motion (RoM) and reducing injury risk (Behm et al., 2021; Konrad et al., 2022; Takeuchi et al., 2024). Three muscle stretching techniques are commonly described in the literature: static, dynamic and pre-contraction stretching (Konrad et al., 2022; Page, 2012). All these techniques acutely increase RoM, which usually lasts up to 30 min. This is mainly due to central mechanisms that increase tolerance to stretch and partially due to an acute reduction in muscle and tendon stiffness (Behm et al., 2016). In this paper, we focus on the effects of the most common type of pre-contraction stretching–proprioceptive neuromuscular facilitation (PNF) stretching (Konrad et al., 2022; Medeiros et al., 2016; Page, 2012). Different PNF methods include contract-relax, hold-relax and contract-relax-antagonist-contract. The muscle contraction, which is part of the stretching, lasts up to 10 s and is usually performed with 75%–100% of maximum voluntary contraction (Konrad, et al., 2022; Page, 2012; Reis et al., 2013). PNF stretching has consistently been shown to increase RoM both acutely and in the long term, with effects comparable to static stretching. However, the role of central mechanisms (e.g., increased stretch tolerance) and peripheral mechanisms (e.g., decreased musculotendinous stiffness) in these effects is not entirely clear (Behm et al., 2023; Borges et al., 2018; Konrad et al., 2024). Increased muscle and tendon stiffness are reported in the literature as potential risk factors for sports injuries, suggesting that PNF stretching could be a potential intervention to mitigate such risks (Konrad, et al., 2022; Place et al., 2013; Reis et al., 2013).

Tissue stiffness is defined as the resistance to deformation under the application of force (Guimarães et al., 2020). Insight into muscle stiffness can be obtained using ultrasound elastography (Guimarães et al., 2020). A specific subtype of this method is shear wave elastography (SWE) (Taljanovic et al., 2017). SWE is a quantitative method that determines the stiffness or elasticity of soft tissue by measuring the speed of propagation of ultrasonic shear waves at precisely defined locations in both superficial and deep tissues (Haueise et al., 2024; Koppenhaver et al., 2022). An ultrasonic pulse generates shear waves in the observed tissue, which propagate parallel to the direction of muscle fibers. The speed of shear wave propagation depends on the stiffness of the observed tissue. This can be expressed in m/s as the actual speed of shear wave propagation, but it is most commonly converted to kPa using Young’s modulus, and, assuming a constant tissue density, converted to the shear modulus (Sigrist et al., 2017; Taljanovic et al., 2017).

Most of the research on PNF techniques to date focuses on its effect on RoM and muscle performance. As mentioned earlier, PNF stretching seems to be equally effective for improving RoM compared to static stretching, both in acute and long-term applications. In their recent review, Konrad et al. (2024) found that all types of stretching (i.e., PNF, static, and dynamic stretching) show positive acute and chronic effects on the range of motion, with no significant differences between the different types of stretching (Konrad et al., 2024). However, the underlying mechanisms behind the increase in RoM are less clear, as it is not entirely understood whether the increases in RoM is primarily due to increased stretch tolerance or a reduction in muscle stiffness. It seems that larger volumes and intensities of stretching can elicit a reduction in muscle stiffness, while RoM increases in cases of lower volume/intensity are primarily due to increased stretch tolerance (Freitas et al., 2018). However, the amount of stretching needed to reduce muscle stiffness is not clearly established (Mizuno, 2017). In addition, different stretching techniques may improve RoM through different mechanisms. In case of the short-term effects, it seems that static stretching is more potent to reduce the stiffness of the gastrocnemius, whereas PNF stretching primarily increases RoM through increase in stretch tolerance (Nakamura et al., 2015; Nakamura et al., 2021). On the contrary, a recent study reported that PNF stretching could acutely reduce the stiffness of the biceps femoris muscle, while static stretching did not (Železnik et al., 2024).

There are few studies that examine the effects of PNF stretching on the rectus femoris muscle (hereafter RF). In two studies, authors reported increased RoM after applying the PNF method (Higgs and Winter 2009; Marek et al., 2005). However, Place et al. (2013) did not report increased RoM following brief stretching of the RF (Place et al., 2013). Konrad et al. (2022) examined the effect of PNF stretching combine with post-stretching dynamic exercises on muscle stiffness of the RF, but observed no changes (Konrad et al., 2022). However, no previous study examined the isolated effect of PNF stretching on RF stiffness. Therefore, the aim of this study was to determine the acute effects of PNF stretching on RF stiffness and to describe the relationship between the volume of stretching and this effect (i.e., the dose-response relationship). We hypothesized that PNF stretching will reduce the stiffness of the RF muscle (compared to passive rest) and the effect will be more pronounced with additional sets of stretching being performed. The RF was selected for assessment due to its functional significance as a biarticular muscle involved in both knee extension and hip flexion, making it a key contributor to many athletic and daily activities. Additionally, its anatomical structure and superficial location are well-suited for reliable SWE measurements. Despite its importance, limited research exists on the acute effects of PNF stretching on RF stiffness, presenting a critical gap this study aimed to address.

## 2 Methods

### 2.1 Participants

A sample of 30 physically active young adult individuals was recruited for the study (23 females; age: 23 ± 2.5 years; body height: 166.2 ± 7.2 cm; body mass: 62.4 ± 7.2 kg; 7 males; age: 23.7 ± 2.7 years; body height: 183.9 ± 6.3 cm; body mass: 85.3 ± 15.5 kg). All participants were healthy, without injuries or any disorders of the lower limb. The recruited sample was based on the study by Železnik et al. (2024), which found a high effect of PNF stretching (3 sets) on hamstring stiffness (η^2^ = 0.33). The calculation suggested that 8 participants per group are sufficient with a statistical power of 90% and an alpha value of 0.05 (sample size for interaction between group and time; G*Power 3.1 software, Heinrich Heine University, Düsseldorf, Germany). Given that this study focuses on a different muscle groups and that responses could be potentially smaller, we decided to increase the sample size to 15 individuals per group. All procedures are in accordance with the ethical approval by the University of Primorska Commission for Ethics in Research Involving Human Subjects (KER UP) (No. 4264–19-6/23). Participants were informed about the experimenting protocol and written consent about participation was required prior to the intervention.

### 2.2 Study design and procedures

Participants were asked not to engage in any form of exercise (i.e., resistance exercise and endurance activities) 48 h prior to the measurements that could affect the increased stiffness of the RF. The intervention was conducted in a single session, which lasted approximately 30 min. The study was conducted in the afternoon hours (12.00–16.00) in an air-conditioned (21.0°C–22.0°C) laboratory. After arriving to the laboratory participants signed the consent form and filled-in a questionnaire including age, body height and mass, involvement in training processes and frequency of resistance training. Afterwards, each participant was randomly assigned to either the intervention or control group by drawing papers from an opaque envelope. Participants were then instructed to rest on physical therapy table for 5 min. After that, SWE measurements were taken on two locations on RF, followed up by the intervention–six sets of PNF stretching protocol. SWE measurements were also taken after second, fourth and sixth set. The visit for each participant, including the familiarization with the procedures, lasted ∼60 min.

### 2.3 Shear-wave elastography

**FIGURE 1:**
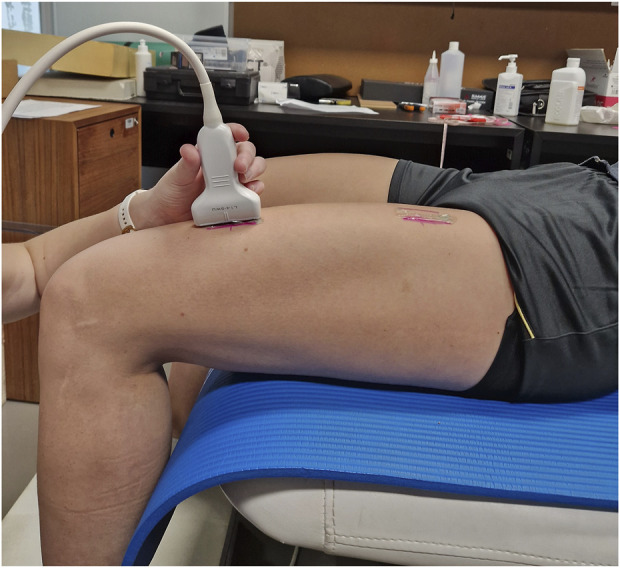
Shear-wave elastography measurements–probe positioning and measurement locations.

**FIGURE 2:**
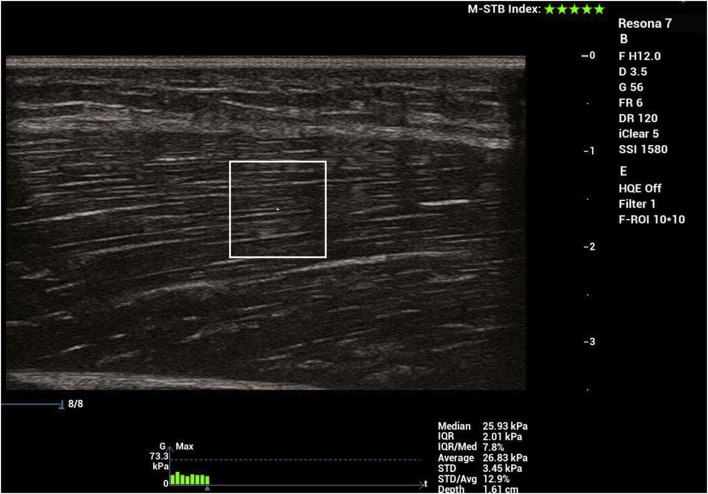
Snapshot of shear-wave elastography measurements.

SWE measurements were performed by a kinesiologist with prior experience in using this technique, ensuring consistency and reliability in data collection. Muscle stiffness was measured using the Resona 7 diagnostic ultrasound system (Mindray, Shenzhen, China) with the shear wave method. A probe (model L14-5WU, Mindray, Shenzhen, China) with a water-soluble hypoallergenic ultrasound gel (AquaUltra Basic–Ultragel, Budapest, Hungary) was used. The measurement location was determined individually for each participant at the 33% and 66% of the distance between superior anterior lilac spine and the apex of the patella. This distance was measured with a tape measure and the locations were marked with a semi-permanent marker to ensure consistency (Figure 1). The region of interest size was set to 1 × 1 cm. The depth of the region of interested was individually determined to ensure that only muscular tissue was encompassed, and was maintained throughout the measurement process (Figure 2). Muscle stiffness was expressed as the shear modulus (in kPa). Data was transcribed into prepared forms during the measurement. The final recorded value of each measurement was the average of eight consecutive measurements. During this time, we determined the proximal and distal points on the left leg where RF stiffness was measured. After the 5 min of lying down, we measured the muscle stiffness. We performed two stiffness measurements first at the distal third and then two measurements at the proximal third. After each measurement, the stiffness data were recorded immediately. The same measurements were performed after the second, fourth and sixth set of PNF stretching (in the case of the control group, after standing for the same time intervals).

### 2.4 Stretching intervention

**FIGURE 3:**
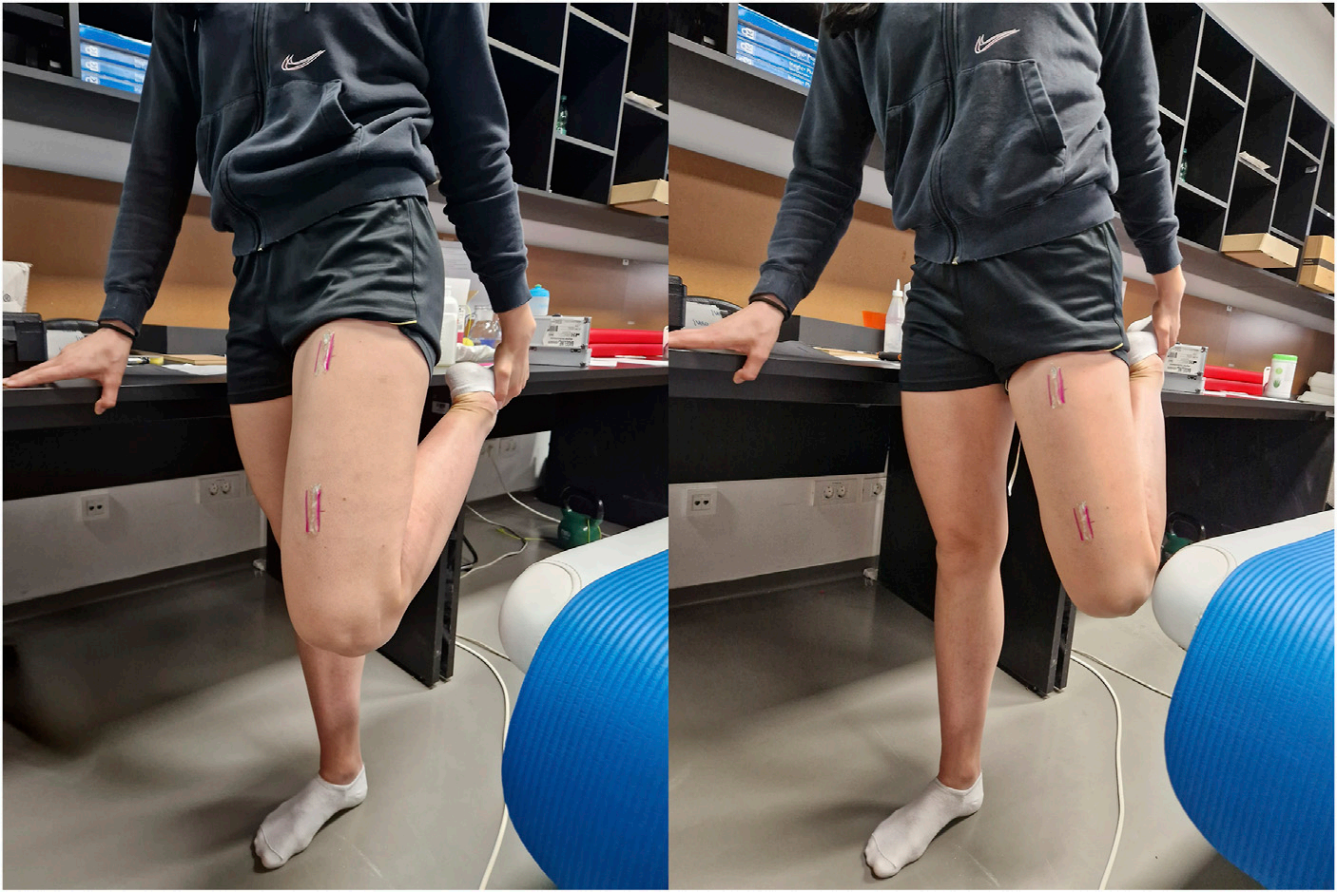
The position for rectus femoris muscle stretching.

Regardless of the group, all participants were lying down for 5 min at the beginning to eliminate the effects of prior movement. The intervention involved six sets of PNF stretching performed on the rectus femoris muscle in a single-leg standing position. Each set included three cycles of 10-s maximal stretches followed by 5-s isometric contractions, with manual resistance applied by the participant. Thus, across six sets, the total time under tension was 135 s, which is within the range of durations reported in previous studies (20–900 s) (Behm et al., 2023). This duration was chosen to balance practicality and participant comfort while adhering to realistic PNF protocols commonly used in athletic and clinical settings. Participants in intervention group were performing PNF stretching in a single-leg standing position (knee flexion), which is commonly used in practice and previous studies as the PNF stretching position for the RF (Konrad et al., 2022; Konrad et al., 2022). When stretching participant stood upright on one leg and pulled the ankle of the opposite leg to the position of maximum knee flexion (Figure 3). Participant had to stretch to the point of discomfort for 10 s, followed by a 5-s maximal contraction in the stretch position against manual resistance provided by themselves. This was repeated three times, resulting in a total duration of 45 s, followed by 2 min of rest (lying down on a physical therapy table). Participants were lying in a supine position with the hip joint in a neutral position, with the knee joint bent over the edge of the table at a 90° angle and with supported feet. Participants performed the PNF stretching six times, with 2 min of lying down between each set in the position as described before. This rest interval was chosen to allow for the measurements of muscle stiffness. Participants in the control group were standing on both feet instead of performing the 45-s PNF stretch.

### 2.5 Statistical analysis

The data are presented as means ± standard deviations. The normality of the data distributions for all variables was verified with the Shapiro-Wilk test and visual inspection of histograms and Q-Q plots. To compare baseline stiffness between groups and gender, independent samples t-tests were utilized. The reliability of the shear modulus measurements between the repetitions was assessed using the Intraclass Correlation Coefficient (ICC). The effects of PNF stretching on muscle stiffness were evaluated using a mixed-design ANOVA. The analysis included one between-subject factor (group: PNF and CG) and two within-subject factors (time: before stretching, after 2 sets, after 4 sets, after 6 sets, and location: distal and proximal). The main effects of group, time, and location, as well as their interactions, were tested to determine the impact of the PNF stretching intervention on shear modulus. Post-hoc tests (Bonferroni correction) were conducted to further explore significant main effects and interactions. The threshold for statistical significance was set at α < 0.05, and all analyses were carried out in SPSS statistical software (version 25.0, IBM, United States of America).

## 3 Results

### 3.1 Baseline outcomes

There were no statistically significant differences between the experimental (11 females, 4 males) and control groups (12 females, 3 males) in terms of age (22.96 ± 2.2 years and 23.3 ± 2.1 years; *p* = 0.668), body height (171.2 ± 6.4 cm and 172.1 ± 6.9 cm; *p* = 0.714) and body mass (66.7 ± 9.4 kg and 67.5 ± 8.5 kg; *p* = 0.809). Before stretching, there were no statistically significant differences between the experimental and control groups regarding stiffness at the distal point (t = 1.4; *p* = 0.171) and at the proximal point (t = 1.72; *p* = 0.095). Likewise, there were no differences at any point between men and women (t = 0.77–0.93; *p* = 0.443–0.468). Before stretching, there was a statistically significant difference (t = 6.7; *p* < 0.001) between the stiffness of the distal point (16.6 ± 3.0 kPa) and the proximal point (23.7 ± 5.2 kPa).

### 3.2 Reliability

Reliability between the repetitions was calculated separately for each of the 8 measurements sets (2 locations and 4 time points). According to the ICC, relative repeatability was moderate for 1 out of 8 measurements (ICC = 0.70), good for 5 out of 8 measurements (ICC = 0.78–0.89), and excellent for 1 out of 8 measurements (ICC = 0.95). However, it is important to note that considering the lower bounds of the confidence intervals, 1 out of 8 measurements exhibits potentially unacceptable relative repeatability (ICC = 0.46), and another 3 measurements show only moderate repeatability (ICC = 0.60–0.65). Typical errors for individual measurements ranged from 1.36 to 1.98 kPa (upper limit of the confidence interval 1.82–2.66 kPa). Coefficients of variation exceeded the 10% threshold in 2 out of 8 measurements.

### 3.3 Effect of PNF stretching interventions

The results at individual time points for both groups, separated for each measurement point, are presented in Table 1.

**TABLE 1:** Descriptive statistics of stiffness measurements.

Time point	Group	Distal (kPa)		Proximal (kPa)	
		Mean	SD	Mean	SD
Before stretching	PNF	15.87	3.30	25.25	5.89
	CG	17.40	2.57	22.02	4.01
After 2 sets	PNF	17.19	4.78	23.27	5.53
	CG	18.24	2.83	23.53	2.79
After 4 sets	PNF	16.67	3.52	24.99	6.76
	CG	17.29	3.64	24.68	6.68
After 6 sets	PNF	17.08	3.57	23.07	3.68
	CG	17.75	3.92	24.18	4.44

**FIGURE 4:**
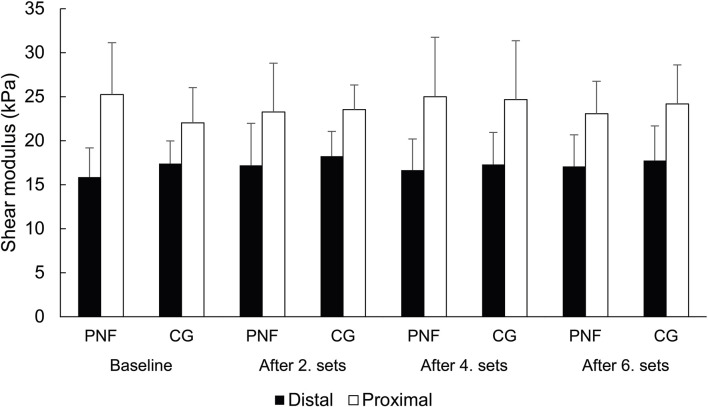
Shear modulus across groups, location and time.

The effect of the group was not statistically significant (F = 0.05; *p* = 0.830). There was also no statistically significant main effect of time (F = 0.545, *p* = 0.653) or interaction between time and group (F = 0.810, *p* = 0.492). The effect of location was statistically significant and substantial (F = 63.6; *p* < 0.001; η^2^ = 0.69), but there was no statistically significant interaction between location and group (F = 0.813, *p* = 0.375) or between time and location (F = 1.504, *p* = 0.220), and no three-way interaction between time, location, and group (F = 2.128, *p* = 0.103). Post-hoc tests indicate that shear modulus was higher on the proximal point, and this difference was independent of the time point of measurement or group (t = 5.7–6.7; all *p* < 0.001). Overall, the results suggest that PNF stretching did not statistically significantly affect muscle stiffness at any measurement point. These findings are presented in Figure 4, illustrating that the differences between distal and proximal locations were consistent across all time points in both groups, while temporal changes within each group were negligible.

## 4 Discussion

The primary objective of this study was to examine the acute effects of PNF stretching on the stiffness of the RF using SWE. Our hypothesis was that PNF stretching would reduce RF stiffness and that this effect would be more pronounced with an increased number of stretching sets. However, our findings indicate that PNF stretching does not significantly reduce RF stiffness compared to passive rest, regardless of the number of stretching sets performed. These results suggest that PNF stretching may not influence RF stiffness acutely, challenging the assumption that this stretching technique can decrease stiffness. This highlights the need for further investigation into muscle-specific effects of PNF stretching.

Prior research has shown that PNF stretching is effective in increasing range of motion (RoM) both acutely and chronically (Behm et al., 2023; Konrad et al., 2024). However, the contribution of different underlying mechanism is not yet fully understood. A recent study by Železnik et al. (2024) reported that PNF stretching significantly reduced the stiffness of the biceps femoris muscle and Nakamura et al. (2015) showed that PNF stretching reduced the stiffness of the medial gastrocnemius muscle, which is in contrast with our results. It is important to acknowledge that the increase in RoM after PNF appears to be predominantly caused by increased stretch tolerance. Stretching may increase pain thresholds by inhibiting nociceptive signals through afferent input from muscles and joints and by promoting enkephalin release in the dorsal horn, reducing nociception transmission (Nakamura et al., 2015; Fukaya et al., 2022). These analgesic effects could enhance stretch tolerance. With these mechanisms playing a crucial role, it can be speculated that changes in tissue stiffness, if any, are smaller and difficult to detect.

It can aslo be speculated that the effects of PNF stretching on muscle stiffness might be muscle-specific. Interestingly, Konrad et al. (2022) found a decrease in vastus medialis but not RF stiffness following PNF stretching combined with post-stretching dynamic exercises. Overall, our results coupled with previous findings suggest RF might be less responsive to PNF stretching in terms of stiffness reduction compared to other muscles like the biceps femoris and medial gastrocnemius muscles. Additionally, Place et al. (2013) observed no significant increase in RoM following brief PNF stretching of the quadriceps, which supports our findings that PNF stretching does not always lead to changes in muscle mechanical properties. The discrepancies between our findings and those of studies reporting stiffness reduction could be attributed to differences in the stretching protocols used. For instance, studies showing significant reductions in muscle stiffness often involve longer durations of stretching or higher intensities (Nakamura et al., 2021). The protocol in our study involved shorter durations of muscle elongation and incorporated relatively long rest periods (120 s), which might have influenced the overall effectiveness of the stretching intervention. In summary, our findings suggest that the effects of PNF stretching on muscle stiffness are not uniform across different muscles and protocols. The lack of significant changes in RF stiffness following PNF stretching in our study reflects the complexity of factors that influence muscle stiffness and the need for further research to understand these mechanisms.

The absence of significant differences in RF stiffness following PNF stretching in this study can be attributed to several key factors related to the method of stretching, particularly the duration of the stretching and the rest periods incorporated. Previous research has suggested there is a dose-response relationship between stretching time and its effectiveness in reducing muscle stiffness (Nakamura et al., 2013; Ryan et al., 2009). Longer durations of stretching have been shown to be more effective in decreasing stiffness, as demonstrated by Nakamura et al. (2021), where 180 s of stretching resulted in a significant reduction in shear modulus. In contrast, the actual muscle elongation time in our study was considerably shorter, with only 45 s of PNF stretching applied. Additionally, the inclusion of a 120-s rest period after every two sets of PNF stretching might have mitigated the stiffness reduction effects observed initially. Mizuno et al. (2013) suggested that the change in muscle stiffness after stretching intervention returned to baseline levels rapidly (Mizuno et al., 2013). Also, Nojiri et al. (2019) showed that longer rest periods during stretching intervention can attenuate the change in muscle stiffness, potentially restoring stiffness levels to their pre-stretching state (Nojiri et al., 2019). This recovery could explain the lack of significant change in muscle stiffness despite multiple sets of PNF stretching.

Moreover, the nature of PNF stretching itself might play a role in the absence of observed stiffness reduction. As discussed by Nakamura et al. (2015), effective reduction of muscle stiffness requires sustained muscle elongation. However, PNF stretching includes phases of muscle contraction, which are contrary to elongation and may counteract the stiffness reduction effects. Given that our PNF stretching protocol involved intermittent muscle contractions, the overall effect on muscle stiffness may have been diminished. In summary, the combined factors of shorter muscle elongation time, the inclusion of rest periods, and the nature of PNF stretching with muscle contractions likely contributed to the absence of significant reductions in RF stiffness in this study. Further research with longer stretching durations and different protocols may be necessary to fully understand the potential of PNF stretching in reducing muscle stiffness.

## 5 Strengths and limitations

This study provides new insights into the acute effects and dose-response relationship of PNF stretching on rectus femoris stiffness, addressing a key gap in the existing research. By using shear-wave elastography, we ensured accurate and reliable measurements of muscle stiffness. The protocol with multiple time points and a thorough reliability analysis, adds confidence to our findings. These results challenge common assumptions about the effectiveness of PNF stretching and highlight the need to consider muscle-specific responses in future research.

This study also has several limitations that should be considered when interpreting the results. A key limitation of this study is the absence of range of motion (ROM) assessments before and after PNF stretching. While we focused on changes in muscle stiffness, ROM measurements could have provided insight into whether increased stretch tolerance occurred despite unchanged stiffness. Future studies should include both stiffness and ROM assessments to better understand the functional implications of PNF stretching. Another limitation of this study is the relatively short total time under stretch (135 s), which may not have been sufficient to induce significant viscoelastic adaptations in the muscle. While this duration aligns with practical PNF protocols, longer stretching durations may yield different outcomes and should be explored in future research. Next, the sample size was relatively small, not gender balanced, and consisted of young healthy adults, which limits the generalizability of our findings. Second, the duration of the stretching and the rest periods may not reflect the common practical application of the PNF technique. The total muscle elongation time was shorter than that in studies that reported significant reductions in muscle stiffness. Additionally, the 120-second rest periods may have allowed for recovery of muscle stiffness, potentially diminishing the effects of the PNF stretching. The study focused exclusively on the rectus femoris muscle. The effects of PNF stretching might vary across different muscles, and our findings cannot be generalized to other muscle groups without further investigation. Further, we did not assess participants’ prior familiarity with PNF stretching, which could have influenced their responses to the intervention. Future studies should consider documenting prior experience to better account for its potential impact on outcomes. Lastly, we used SWE as the sole method for assessing muscle stiffness. While this method is reliable and non-invasive, it may not capture all aspects of muscle mechanical properties. Complementary methods could provide a more comprehensive understanding of musculotendinous stiffness changes following PNF stretching.

## 6 Conclusion

In conclusion, this study found that PNF stretching does not acutely reduce the stiffness of the rectus femoris muscle when compared to passive rest. The inclusion of long rest intervals between stretching sets may have contributed to this lack of significant changes in muscle stiffness. These results challenge the assumption that PNF stretching universally decreases muscle stiffness and underscore the complexity of factors influencing muscle properties. Further research is necessary to explore the acute chronic effects of PNF stretching, investigate different muscle groups, and understand the underlying mechanisms affecting muscle stiffness.

## Data Availability

The raw data supporting the conclusions of this article will be made available by the authors, without undue reservation.
